# The Role of Nutrition in the Prevention and Intervention of Type 2 Diabetes

**DOI:** 10.3389/fbioe.2020.575442

**Published:** 2020-09-15

**Authors:** Yajie Guo, Zihua Huang, Dan Sang, Qiong Gao, Qingjiao Li

**Affiliations:** The Eighth Affiliated Hospital, Sun Yat-sen University, Shenzhen, China

**Keywords:** nutrition, diet, genetics, epigenetics, gut microbiota, type 2 diabetes

## Abstract

Type 2 diabetes (T2D) is a rapidly growing epidemic, which leads to increased mortality rates and health care costs. Nutrients (namely, carbohydrates, fat, protein, mineral substances, and vitamin), sensing, and management are central to metabolic homeostasis, therefore presenting a leading factor contributing to T2D. Understanding the comprehensive effects and the underlying mechanisms of nutrition in regulating glucose metabolism and the interactions of diet with genetics, epigenetics, and gut microbiota is helpful for developing new strategies to prevent and treat T2D. In this review, we discuss different mechanistic pathways contributing to T2D and then summarize the current researches concerning associations between different nutrients intake and glucose homeostasis. We also explore the possible relationship between nutrients and genetic background, epigenetics, and metagenomics in terms of the susceptibility and treatment of T2D. For the specificity of individual, precision nutrition depends on the person’s genotype, and microbiota is vital to the prevention and intervention of T2D.

## Introduction

Diabetes mellitus, previously considered as a disease of minor significance to health, is now becoming one of the main threats to human health both in developed and developing countries (Zimmet et al., 2001). There has been an explosive increase in the number of people diagnosed with diabetes in recent decades worldwide (King et al., 1998). According to the ninth edition of the IDF Diabetes Atlas in 2019, 488 million adults aged 20–99 years live with diabetes in the world, and the number will reach 578 million by 2030 and 700 million by 2045. It is estimated that 4.2 million adults aged 20–79 years will die of diabetes, which accounts for 11.3% of all deaths. And this is equivalent to eight deaths every minute.

Diabetes is defined as a metabolic disease characterized by persistent hyperglycemia caused by multiple factors including genetics, nutrition, environment, and physical activity. There are two main forms of diabetes, type 1 diabetes and type 2 diabetes (T2D) (Alberti and Zimmet, 1998). T2D accounts for more than 90% of all diabetes cases (Zimmet et al., 2001), and the diabetes epidemic particularly relates to T2D. Insulin resistance and/or abnormal insulin secretion are the main characters of T2D. Apart from the heightened genetic susceptibility of ethnic groups, environmental and behavioral factors are also very important in the development of T2D. Globalization results in altered dietary and lifestyle habits (Malik et al., 2013), such as taking more high-fat or high-carbohydrate foods and sedentary lifestyles with low energy expenditure (Zimmet et al., 2001). Diets induce multiple metabolic processes and modify the metabolism homeostasis of the organism (Manore et al., 2017). Therefore, unhealthy dietary habits such as Western diet have been one of the most important drivers of glucose metabolism disorder that leads to diabetes finally (Rico-Campa et al., 2019).

The increase in the prevalence of T2D is associated with a concomitant rise in the incidence of metabolic disorders. Long-term high glucose levels will trigger chronic metabolic syndrome and include obesity (Schwartz and Porte, 2005), cardiovascular disease, retinopathy, nephropathy, dyslipidemia, and hypertension (Moller, 2001). T2D now represents a risk of coronary heart disease, and nearly 80% of diabetic mortality is diabetes-induced cardiovascular disease (Haffner et al., 1998). The life qualities of patients with diabetes decrease largely for the serious diabetes complications.

Diet alone or with hypoglycemic agents is the way to control blood glucose levels in the treatment of T2D (Zimmet et al., 2001; Ley et al., 2014). Different diets with varied nutrient composition result in changes of metabolites and gut microbiome that are responsible for the glucose metabolism of the whole body (Qin et al., 2012; Guasch-Ferre et al., 2016). For example, different amino acid content diets can lead to alterations of plasma branched-chain amino acid (BCAA) concentrations, which are linked to the risk of T2D (Garcia-Perez et al., 2017). Fiber- and protein-enriched diet changed the abundance of *Akkermansia muciniphila*, decreasing fasting glucose levels of participants (Dao et al., 2016). However, the interactions between dietary and glucose metabolism need further study to understand the importance of its actions for glucose management. It is important to identify and make suitable dietary solutions that can diminish the prevalence of diabetes and its related complications (San-Cristobal et al., 2015). These include different kinds of food and also healthy dietary habits.

Genome-wide association studies (GWASs) have revealed many genetic variants related to the susceptibility of complex diseases, and moreover, the interactions between genetic information and nutrition are attracting more attention recently, namely, nutrigenetics. Because of the genetic variability between individuals, the responses to dietary are different. Also, the specific diet and nutrition modify gene expression, epigenetic features, and gut microbiome to personalize the response to interventions. This prompts us to explore more possibilities to understand the pathophysiological mechanisms and precision nutrition solutions to prevent and manage T2D more efficiently.

Herein, first, we introduce the major metabolic pathway related to T2D, namely, insulin signaling pathway, and the compounding factors as well. Then, the roles of macronutrient, micronutrient, and other chemicals in maintaining metabolic homeostasis of the body and their effects on T2D are reviewed in detail. In addition, some nutritional recommendations for T2D are summarized. From the perspective of precision nutrition, we review the diet interactions with genetic background, epigenetics, and gut microbiota contributing to the risk of T2D. Also, responses to dietary interventions mainly aiming at weight loss and management of insulin resistance are screened for their interaction with genetic, epigenetic features, and gut microbiota.

## Regulation of Glucose Metabolism

**FIGURE 1:**
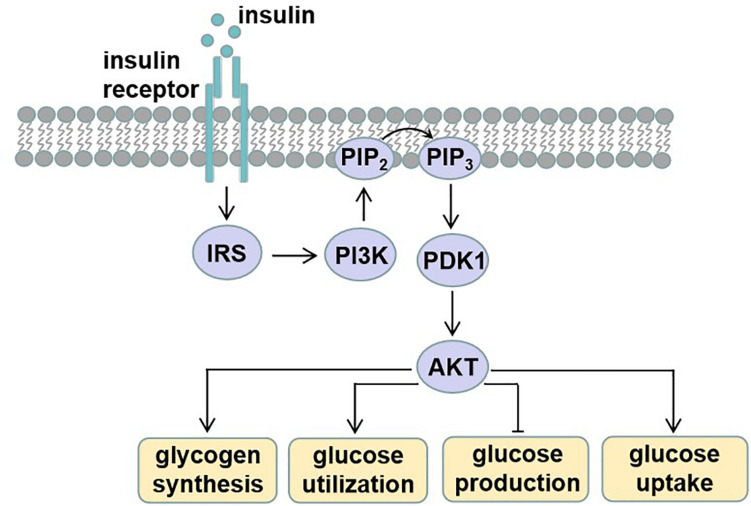
Insulin signaling. Insulin binds and activates insulin receptor (INSR), causing phosphorylation of insulin receptor substrate (IRS). Tyrosine phosphorylated IRS proteins recruit phosphatidylinositide-3 (PI3K), which catalyzes the production of phosphatidylinositol-3,4,5-tris-phosphate (PIP_3_) from PIP_2_. PIP_3_ then recruits proteins with PH domains such as pyruvate dehydrogenase kinase 1 (PDK1), which phosphorylates activating protein kinase B (AKT). These effector proteins mediate the effects of insulin on glucose production, utilization, and uptake, as well as glycogen synthesis.

Circulating blood glucose is derived from diet via intestinal absorption, and the process of glucose production is called gluconeogenesis and glycogen breakdown (Rines et al., 2016). Current therapeutic approaches to treat T2D rely on the molecular signaling pathways and targets that impair glucose homeostasis. Insulin signaling pathway dysregulation or insulin resistance is the main reason for T2D. Insulin is an endocrine peptide hormone secreted by the pancreas, and it binds to membrane-bound receptors in target cells of liver, adipose tissue, and skeletal muscle to trigger metabolic responses to numerous stimuli (Petersen and Shulman, 2018). Insulin exerts its low glucose function by binding to the insulin receptor (INSR), and then activated INSR recruits phosphotyrosine-binding scaffold proteins such as the INSR substrate (IRS) family. IRS proteins have NH_2_-terminal pleckstrin homology (PH) and PTB domains that target them to activate INSR. Then, the tyrosine phosphorylated IRS proteins recruit PI3K heterodimers that contain a regulatory p85 subunit and a catalytic p110 subunit. PI3K catalyzes the production of phosphatidylinositol-3,4,5-tris-phosphate (PIP_3_) from PIP_2_ and PIP_3_ and then recruits proteins with PH domains to the plasma membrane, such as pyruvate dehydrogenase kinase 1, which directly phosphorylates AKT. The activated AKT phosphorylates many downstream substrates in various signaling pathways, making it a key node in insulin signaling (Petersen and Shulman, 2018). The activated insulin signaling decreases glucose production, increases glycogen synthesis, and also increases glucose uptake into peripheral tissues such as skeletal muscle and adipose tissue (Figure 1).

**FIGURE 2:**
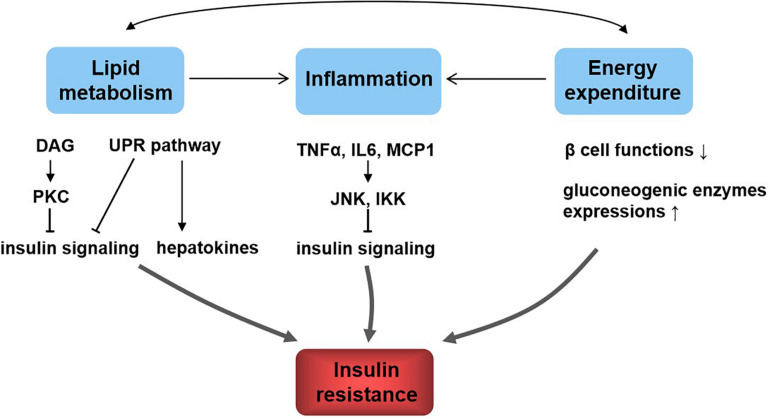
Relationship between lipid metabolism, energy metabolism, inflammation, and insulin resistance. Lipid metabolism and energy metabolism disorder lead to inflammation and affect each other. These all contribute to insulin resistance. The underlying mechanisms include diacylglycerol (DAG), activate protein kinase C (PKC) and lipid accumulation, trigger the unfolded protein response (UPR) pathway, and result in insulin signaling inhibition; UPR affects hepatokine secretion to induce insulin resistance; inflammatory molecules such as tumor necrosis factor α (TNF-α), interleukin 6 (IL6), and monocyte chemotactic protein 1 (MCP1) activate c-Jun N-terminal kinase (JNK) and IκB kinase (IKK), which in turn impair insulin signaling; energy homeostasis disorder impairs β-cell functions, reduces PI3K signaling, and enhances gluconeogenic enzyme expressions, resulting in insulin resistance.

The dysfunction of insulin signaling will cause insulin resistance, which is a complex metabolic disorder that is closely linked to many pathways including lipid metabolism, energy expenditure, and inflammation (Figure 2). Hepatic lipid accumulation is known to cause insulin resistance (Samuel and Shulman, 2012). Diacylglycerol species activate protein kinase C (PKC), which results in impaired insulin signaling (Perry et al., 2014). An excess of lipid accumulation in liver is often accompanied by hepatic inflammation. Kupffer cells and macrophages will decrease insulin sensitivity by secreting proinflammatory molecules, which activate serine/threonine kinases such as c-Jun N-terminal kinase (JNK) and IκB kinase that in turn impair insulin signaling (Lackey and Olefsky, 2016). Moreover, lipid accumulation triggers the unfolded protein response (UPR) pathway, which impairs insulin signaling (Ozcan et al., 2004). UPR may also alter hepatokine secretion and consequently contribute to the development of insulin resistance (Koska et al., 2008). Energy expenditure disorder leads to obesity and insulin resistance, because non-esterified fatty acids impair β-cell functions, reduce PI3K signaling, and enhance gluconeogenic enzyme expressions (Kahn et al., 2006). What is more, increased release of tumor necrosis factor α (TNF-α), interleukin 6 (IL6), and monocyte chemotactic protein 1 are all found to be responsible for the development of insulin resistance (Kahn et al., 2006). In addition, hepatokines, proteins produced from liver and secreted into the circulation, also play important roles in regulating insulin signaling (Meex and Watt, 2017). Retinol-binding protein 4 (RBP4), α2-macroglobulin (A2M), fetuin A (FETUA), fetuin B (FETUB), hepassocin (FGL1), leukocyte cell–derived chemotaxin 2 (LECT2), and selenoprotein P (SELENOP) are negative regulators of insulin sensitivity, and they will cause insulin resistance, whereas fibroblast growth factor 21 (FGF21), sex hormone–binding globulin (SHBG), adropin, and angiopoietin-like protein 4 (ANGPTL4) are positive regulators (Lai et al., 2008; Meex and Watt, 2017).

## Macronutrient and T2D

### Carbohydrate

It needs a precise control of glucose metabolism to maintain metabolic homeostasis of the body. Hormonal regulation and the related enzyme transcription induced by different metabolites in response to glucose availability are mainly responsible for the control. Insulin induces INSR autophosphorylation and then recruits and phosphorylates IR substrates 1 and 2 (IRS1/2). This results in phosphatidylinositide-3, 4, 5-P3 (PIP3) production, and activating protein kinase B (PKB/AKT) (Saltiel and Kahn, 2001). Thus, it promotes glucose uptake by different tissues, including liver, adipose tissue, and skeletal muscle; inhibits hepatic glucose output; increases glycogen synthesis; and decreases glycogen decompose (Zhang et al., 2009). Insulin induces anabolic responses such as ribosome biogenesis and protein synthesis, which are dependent on nutritional state. The mTOR/S6K1 signaling pathway is also activated by insulin, which plays a vital role in the regulation of glucose homeostasis (Um et al., 2006). Glucose released by diet stimulates the production of PI3P, recruiting proteins to endosomal membranes and finally activating mTOR/S6K1 signaling pathway (Um et al., 2006).

**FIGURE 3:**
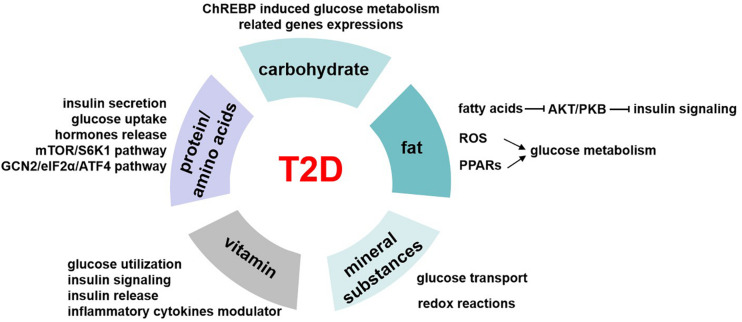
The role of nutrients in T2D. Carbohydrates regulate glucose homeostasis through carbohydrate-responsive element-binding protein (ChREBP) induced glucose metabolism–related genes expressions. Fatty acids inhibit AKT/PKB activation and therefore impair insulin signaling pathway. Besides, fat induces reactive oxygen species (ROS) generation in mitochondrial and activates peroxisome proliferator-activated receptors (PPARs), and all these mediate the regulation of fat on glucose metabolism. The possible underlying pathways or mechanisms of protein/amino acids affecting glucose levels include insulin secretion, glucose uptake, hormone release, mTOR/S6K1 signaling pathway, and GCN2/eIF2α/ATF4 transduction pathway. Mineral substances are activating cofactors and coenzymes for metabolism control, oxidative stress, and genetic transcription. This makes them play roles in glucose transport and redox reactions, which finally affect glucose homeostasis. Vitamin has a role in regulating glucose utilization, insulin signaling, and insulin release from β cells to maintain blood glucose levels, and it is also the modulator of inflammatory cytokines related to glucose metabolism.

Glucose homeostasis involves different pathways that are carried out in part by the transcriptional control of related genes. Carbohydrate induces the expressions of these enzymes, including pyruvate kinase, glucokinase, ATP citrate lyase, and acetyl CoA carboxylase (Haro et al., 2019). And these genes are regulated by the carbohydrate-responsive element-binding protein (ChREBP) (Figure 3), which is a helix–loop–helix leucine zipper transcription factor (Lee and Cha, 2018). It plays a very important role in sugar-induced lipogenesis and glucose homeostasis by regulating carbohydrate digestion and transport (Yamashita et al., 2001). In response to glucose, ChREBP forms a heterodimer and activates the target genes transcriptions, which contain carbohydrate response element motifs. Except for its glucose sensor role, ChREBP is also essential for fructose induced lipogenesis in liver and intestine possibly via the ChREBP-FGF21 signaling axis (Fisher et al., 2017).

Carbohydrate foods that promote sustained but low glucose levels may have benefits to metabolic control of diabetes and its complications. Diets with slow-release carbohydrates lower the glucose and insulin responses throughout the day and improve the capacity for fibrinolysis, which may be a potential therapy to T2D (Russell et al., 2016). When syrup is included in a diabetic diet, it is good to consider sucrose rather than fructose (Wheeler and Pi-Sunyer, 2008). In a short-term trial of T2D patients, scientists showed that isocaloric fructose replacement of other carbohydrates such as sucrose and starch improved glycemic control and had no effects on insulin signaling (Cooper et al., 2012). However, it should take more account to the point that high sucrose or fructose diet is not recommended to diabetic individuals and others who have impaired glucose metabolism. Besides, certain diet components may affect the regulation role of foods on glucose levels. For example, diet with fiber, certain proteins, or lipids to mix may influence the rates of carbohydrate digestion and absorption, which may be beneficial to T2D patients (Russell et al., 2016). Polyols have been used as sugar replacers for about 80 years. Clinical trial showed that polyols had a role in lowering serum glucose levels in T2D patients (Mohsenpour et al., 2019), which may provide a new strategy to manage T2D. Dietary fibers, which are mainly found in cereals, fruits, vegetables, or legumes, showed close associations with T2D. Increased fiber intake, especially soluble fiber, played a beneficial role in improving glycemic control in patients with T2D (Chandalia et al., 2000).

### Fat

Our body obtains kinds of lipid metabolites from diet intake directly or generated intracellularly by liver and adipose tissue in different pathways. Lipidomics help us better understand the circulating lipid species. Among these, some are considered as biomarkers related to insulin resistance, such as stearic acid and deoxysphingolipids, and saturation and chain length of fatty acids (Meikle and Summers, 2017). High-fat diet–induced insulin resistance and T2D have been largely known since 20 years ago. High-fat diet increases lipid accumulation in cells and leads to obesity. Excess of fat increases proinflammatory cytokines and other hormones or factors involved in insulin resistance (Kahn et al., 2006). Free fatty acids inhibit Akt/PKB activation, thus impairing the insulin signaling pathway (Bruce and Febbraio, 2007). Besides, the reactive oxygen species generation in mitochondrial is increased, which also affects the glucose homeostasis (Bruce and Febbraio, 2007). Peroxisome proliferator-activated receptors (PPARs) function as lipid sensors that can be activated by both dietary fatty acids and their derivatives. PPARs regulate the expression of genes involved in a variety of processes including glucose and lipid metabolism, immune response, and cell growth (Evans et al., 2004; Figure 3). PPARα is vital in regulating fatty acid oxidation and therefore has indirect effects on improving glucose metabolism (Evans et al., 2004). Besides, PPARα activates tribbles pseudokinase 3 (TRB3), a direct target, to inhibit AKT activation and impairs insulin sensitivity (Du et al., 2003). PPARγ is an effector of adipogenesis via C/EBP and is responsible for the glucose regulation.

Scientists investigated the impact of fatty acid intake on blood glucose and insulin in the diet of adults with T2D and found that replacement of saturated fats with monounsaturated fatty acids (MUFAs) or polyunsaturated fatty acids may improve their glucose or insulin tolerance (Russell et al., 2016). And *in vitro* experiments have also confirmed that MUFAs or oleate rather than palmitate prevents insulin resistance (Gao et al., 2009). Postprandial hyperlipidemia is common in T2D patients, and it was shown that omega-3 fatty acids could reduce postprandial lipids but may not correct them completely (Tomlinson et al., 2020). However, the role of *trans*-fats in regulating glucose control is still controversial. Meta-analysis showed cholesterol-rich diet had a positive relationship with T2D risk (Tajima et al., 2014). Besides, supplementation of plant sterols or stanols lowered serum cholesterol levels (Derosa et al., 2018) that may be indirectly beneficial to glucose metabolism.

### Protein

Dietary proteins are vital to life for its important role in acquiring essential amino acids to maintain protein synthesis and degradation and supporting cellular processes such as cell growth and development (Tremblay et al., 2007). In recent years, more and more studies have shown that proteins had different effects on glucose homeostasis by affecting insulin action and secretion except for body weight and feeding behavior. In normal or diabetic humans, dietary proteins stimulate insulin secretion so as to reduce glycemia (Spiller et al., 1987). High-protein diets seem to have beneficial effects on weight loss and glucose metabolism, significantly increase insulin sensitivity, and decrease inflammation in the short term (Russell et al., 2016). But long-term high-protein intake seems to result in insulin resistance in the whole body, by increasing mTOR/S6K1 signaling pathway and stimulating gluconeogenesis and high glucagon turnover (Linn et al., 2000). Studies showed that a 6-month high-protein diet (1.87 ± 0.26 g protein/kg body weight per day) in healthy individuals increased fasting glucose levels, impaired hepatic glucose output suppression by insulin, and enhanced gluconeogenesis (Linn et al., 2000). On the other hand, low-protein diets (5%–10% protein calories) suggested improved insulin sensitivity that is beneficial to T2D, and this may be realized through the general control non-derepressible 2 (GCN2)/transcription factor 4 (ATF4)/FGF21 signaling pathway (Haro et al., 2019; Figure 3).

Soy protein is one kind of protein that is good for its hypolipidemic and hypocholesterolemic benefits in humans (Anderson et al., 1995). Studies showed that soy protein intake can positively affect glucose metabolism in addition to its effect on decreasing serum lipids. In comparison with casein, soy protein reduced fasting glucose and insulin levels in animals and prevented insulin resistance induced by a high-sucrose diet. Moreover, in humans, it was also revealed that soy protein decreased glucose levels compared to casein (Hubbard et al., 1989). And this function might be explained by the differential hormonal response. Besides, soy protein can also stimulate INSR mRNA expression and thereby increase insulin signaling in fat and liver finally improve insulin sensitivity in these tissues (Iritani et al., 1997).

Fish protein is another protein and widely known protein for years, as Alaska and Greenland populations have a low incidence of T2D for taking large amounts of fish. In lean fish, protein is the most abundant nutrient; consumption of fish protein showed improved cholesterol transport via high-density lipoprotein and reduced triglycerides via very low-density lipoprotein (Chen et al., 2020). Meanwhile, compared to casein-fed animals, cod protein–fed rats were protected against insulin resistance induced by sucrose or in saturated fat (Lavigne et al., 2000) by stimulating glucose uptake by skeletal muscle (Lavigne et al., 2001). Cod protein activated PI3K/AKT signaling pathway and selectively improved GLUT4 translocation to the T tubules, improving glucose transport in response to insulin (Tremblay et al., 2003). Moreover, human studies also showed cod protein exerted beneficial effects to T2D. Cod protein induced a lower insulin-to-glucose ratio compared with milk protein (von Post-Skagegard et al., 2006) and increased postmeal plasma insulin concentrations compared with beef protein (Tremblay et al., 2007).

Protein breakdown or synthesis leads to the change of amino acids levels. There are eight amino acids that cannot be produced inside the body but must come from food. Amino acids are considered as gene expression regulators such as CHOP, which is important to glucose metabolism (Tremblay and Marette, 2001). Amino acids activate the mTOR/S6K1 pathway, and the activation of mTOR inhibits PI3K that results in insulin resistance (Kimball and Jefferson, 2006). BCAAs are kind of important amino acids in regulating homeostasis. BCAAs regulate the release of hormones, including leptin (LEP), GLP-1, and ghrelin, which affects glucose control (Potier et al., 2009). Besides, BCAAs regulate glucose metabolism partly through activating the mTORC1/PKC signaling pathway (Vary and Lynch, 2007).

A healthy and balanced diet should meet all the requirements in amino acids and proteins from varied sources in appropriate proportions. The canonical pathway to respond to amino acid deficiency is amino acid response (Chou et al., 2012). When the essential amino acids decrease, it would cause the deacetylation of the corresponding tRNAs. Uncharged tRNAs bind and activate the GCN2 kinase, and then the activated GCN2 phosphorylates the eukaryotic initiation factor 2α (eIF2α), and induces ATF4 activation (Hao et al., 2005). Numerous studies have shown that increasing dietary levels of BCAAs had a positive effect on T2D (Lynch and Adams, 2014), whereas others suggested that deficiency of BCAAs was beneficial for improving insulin sensitivity and glucose tolerance. Leucine deprivation or methionine deficiency all showed improved insulin sensitivity, energy expenditure, and thermogenesis via GCN2/eIF2α/ATF4/FGF21 transduction pathway (Haro et al., 2019).

## Micronutrient and T2D

### Mineral Substances

As micronutrient, mineral substances are required at very low concentrations for the normal growth but play important roles in maintaining metabolism homeostasis (Shenkin, 2006). Some of the mineral substances are activating cofactors and coenzymes for metabolism control, oxidative stress, and genetic transcription. The deficiency of mineral substances was shown to have relationship with T2D (Figure 3).

Selenium is a vital component of enzymes for redox reactions such as glutathione peroxidase and thioredoxin reductase in human body, and importantly, the dose range to toxicity is very narrow (Sun et al., 2013). The main dietary sources of selenium are cereals, black tea, milk, mushrooms, soybeans, bamboo shoots, nuts, and broccoli (Rayman et al., 2008). Appropriate concentration of selenium intake can act as an insulin minetic to attenuate diabetes, with the role of decreasing glucose and insulin tolerance, thus preventing hepatic insulin resistance (Zhou et al., 2013). However, high selenium concentration will result in gluconeogenesis, and fasting blood glucose (FBG) levels increased and therefore have a risk to diabetes (Ogawa-Wong et al., 2016).

Vanadium is common in nature but appears at very low concentrations in humans. It occurs with proteins such as transferrin, albumin, and hemoglobin that are vital to the physiological processes (Pessoa and Tomaz, 2010). *In vitro* and *in vivo* researches suggested that vanadium had insulin-mimetic properties and may be a potential therapeutic agent to T2D (Domingo and Gomez, 2016). Oral administration of 1 mg/kg per day of vanadyl sulfate for 4 weeks significantly decreased glucose levels in diabetes patients. The possible mechanism underlying this might be through increasing GLUT translocation to plasma membrane and then resulting in glucose transport increase (Cohen et al., 1995).

Chromium plays an important role in glucose metabolism by enhancing the binding of insulin to INSR (Cefalu and Hu, 2004). Clinical trials suggested 4 months’ supplementation of chromium significantly decreased postprandial and also fasting glucose levels. Mechanisms underlying this beneficial function of chromium may partly be explained by the increase of GLUT2 expression and the activation of PI3K/AKT pathway in skeletal muscle (Panchal et al., 2017).

Zinc is an important component of enzymes that play vital roles in regulating insulin sensitivity and glucose homeostasis. Researches showed that, in patients with T2D, the concentrations of zinc in plasma and tissues are lower (Russell et al., 2016). Zinc supplementation improved insulin sensitivity and glucose tolerance in diabetic mice models (Chen et al., 2000) and was found to have similar functions in humans (Russell et al., 2016).

High sodium intake leads to a higher risk of hypertension and cardiovascular diseases in patients with diabetes mellitus. Sodium intake increases natriuresis via PPARδ/SGLT2 pathway and subsequently regulates glucose metabolism of type 2 diabetic patients (Zhao et al., 2016). In contrast, another substance, magnesium, was suggested to decrease the risk of cardiovascular diseases in T2D patients. Magnesium deficiency was associated with diabetes risk, whereas magnesium supplementation could attenuate insulin resistance and improve glycemic control in T2D patients (Wa et al., 2018).

### Vitamin

In recent years, vitamin has received increased attention because of its roles in regulating the development of T2D by modulating insulin resistance and pancreatic β-cell functions (Figure 3). Among these, vitamins D and E are the two most popular types. Vitamin D was used to be a regulator of bone metabolism but was found to have various clinical functions. It is a key hormone involved in calcium and phosphorous balance with several derivatives (Muscogiuri et al., 2017). Vitamin D receptor (VDR) is found in the pancreatic β cells and insulin response tissues such as skeletal muscle and adipose tissue (Fan et al., 2016). Studies showed that vitamin D affected glucose utilization in VDR-dependent manner in muscle and adipose tissue and activated PPARδ, which is a transcription factor involved in fatty acid metabolism (Grammatiki et al., 2017). Besides, vitamins modulated insulin action and insulin sensitivity by directly stimulating INSR gene expressions (Maestro et al., 2000) or altered calcium flux to influence insulin release of β cells (Muscogiuri et al., 2017). Moreover, vitamin is a negative modulator of inflammatory cytokine such as TNF-α and IL6, which are closely related to insulin resistance (Garbossa and Folli, 2017).

Insulin resistance is the main diagnosis in most T2D patients, and vitamin D deficiency was found to result in insulin resistance and metabolic syndrome such as hypogonadotrophic, renal diseases and cardiovascular complications (Garbossa and Folli, 2017). And some beneficial effects of vitamin D supplementation have been reported. In several clinical trials, vitamin D administration decreased serum fasting glucose levels and improved Homeostatic Model Assessment of Insulin Resistance index in T2D patients (Talaei et al., 2013; Grammatiki et al., 2017). Vitamin E is a fat-soluble vitamin, which is well known for its antioxidant capacity. Besides, it also functions on cell cycle, cell signaling, lipid metabolism, and inflammation (Gray et al., 2011). Several years ago, vitamin E has been reported to have a role in regulation of insulin sensitivity (Galmes et al., 2018). Vitamin E supplementation significantly decreased plasma glucose and hemoglobin A_1__c_ (HbA_1__c_) levels (Paolisso et al., 1993). The underlying mechanisms may include several pathways. For its antioxidant capacity, vitamin E alters IRS1 phosphorylation, thus affecting insulin signaling (Gray et al., 2011). Besides, vitamin E was shown to directly regulate gene expression such as PPARγ, which plays important roles in insulin sensitivity (Landrier et al., 2009).

## Other Chemicals and T2D

Besides macronutrients and micronutrients, others such as phytochemicals and bioactives that are widely distributed in diets or chemicals (such as alcohol) also have potential effects on T2D. Phytochemicals or bioactives exist in fruits, flowers, wood, seeds, bark, and stems, and some of them are found in traditional Chinese medicine (Zhao et al., 2019). They have been reported for their beneficial and therapeutic roles on diabetes in various studies. Phytochemical compounds such as lignans or flavonoids protect against oxidative stress and help diabetic wound healing (Bacanli et al., 2019). Bioactives, such as curcumin, capsaicin, berberine, celastrol, or artemisinin, were shown to improve insulin sensitivity to combat diabetes (Zhao et al., 2019). Despite the promising benefits, the molecular activity and toxicity of these numerous phytochemicals and bioactives need to be explored in further studies.

Alcohol is closely related to diseases such as fatty liver, cardiovascular diseases, and also T2D. Recently, a dose-response meta-analysis suggested that light and moderate alcohol intake may reduce the risk of T2D, whereas heavy alcohol intake showed inconclusive association (Knott et al., 2015).

## Nutritional Recommendations for T2D

Prospective studies and clinical trials suggest different nutritional recommendations for the prevention and management of T2D. And they all highlight the importance of dietary habits and lifestyles. For example, calorie restriction and exercise are helpful to reduce the risk of T2D. From the perspective of nutrients, the quality is more important than the quantity. To better improve glucose control in T2D patients, diets rich in fruits, vegetables, legumes, and whole grains are recommended. Low-carbohydrate, low-GI (glycemic index), and high-protein diet patterns will protect us from hyperglycemia incidence. Moreover, moderate consumption of nuts and alcohol is also beneficial (Ley et al., 2014). Different populations or individuals have different foods, dietary habits, and disease susceptibility as well, so nutritional strategies should vary according to their cultures and genetic background.

## Diet With Genetics, Epigenetics, and Metagenomics Involved in the Risk of T2D

Genetic backgrounds and environments (e.g., high-fat and high-energy dietary habits, and a sedentary lifestyle) are major factors that contribute to high susceptibility of T2D. The impressive progress of next-generation sequencing (NGS) technology has enabled genome sequencing to be obtained in a cheap and reliable large-scale manner, which provides a comprehensive description of genetic variants including single-nucleotide polymorphisms (SNPs), copy number variations, and other structural variants. Various technologies combined with NGS are developed to explore an increasingly diverse range of biological problems extensively for transcriptome, epigenome, and microbiome. Genetic variants account for only 5–10% for the observed heritability of T2D (Schwenk et al., 2013). Recent advances in precision nutrition have recognized that an individual’s diet may increase the disease risk of T2D by interacting with specific gene variants, affecting the expression of genes, modifying the epigenetic features, or altering microbial composition involved in critical metabolic pathways.

### Genetic Variants and Diet Interactions

Genetic variants are the most widely studied features in the field of precision nutrition, and the GWASs have generated extensive knowledge about the genetic background of T2D (Table 1; Hindy et al., 2012; Ortega-Azorin et al., 2012; Ericson et al., 2013; Hwang et al., 2013; Ouhaibi-Djellouli et al., 2014; InterAct Consortium, 2016; Park et al., 2017; Schuler et al., 2017). For example, The α-ketoglutarate–dependent dioxygenase (FTO) and melanocortin-4 receptor (MC4R) genes were confirmed to be obesity-associated loci, which promotes researchers to study the association of these variants with T2D. GWASs showed these two genes were not significantly associated with diabetes, and conversely, SNP–diet interactions were found to play an important role in the risk of T2D (Ortega-Azorin et al., 2012). FTO rs9939609 and MC4R rs17782313 polymorphisms conferred a higher risk of T2D in subjects with low adherence to the Mediterranean diet. Transcription factor 7–like 2 protein (TCF7L2) was reported to play an important role in the pathogenesis of T2D, and the rs7903146 polymorphism was associated with a high risk of T2D in an Algerian population (Ouhaibi-Djellouli et al., 2014). In addition, the risk was increased in the subjects with both rs7903146 SNP and high dessert and milk intakes, which had higher fasting plasma glucose concentration.

**TABLE 1:** SNPs–diet interactions increase the risk of type 2 diabetes.

Genes	Polymorphisms	Alleles	Diet interaction	References
FTO	rs9939609	T	Low adherence to Mediterranean diet	Ortega-Azorin et al., 2012
MC4R	rs17782313	T	Low adherence to Mediterranean diet	Ortega-Azorin et al., 2012
TCF7L2	rs7903146	T	High dessert and milk	Ouhaibi-Djellouli et al., 2014
TCF7L2	rs7903146	T	High fiber	Hindy et al., 2012
IRS1	rs2943641	T	Low carbohydrate intake	Ericson et al., 2013
Adiponectin gene	SNP276G > T	G > T	Carbohydrate	Hwang et al., 2013
TCF7L2	rs12255372	T	Coffee	InterAct Consortium, 2016
PGC-1α	rs10517030/	C/T	Low-energy diet	Park et al., 2017
	rs10517032			
ACE gene	rs4343	I/D	High-fat diet	Schuler et al., 2017

*FTO, FTO α-ketoglutarate–dependent dioxygenase; MC4R, melanocortin 4 receptor; TCF7L2, transcription factor 7–like 2.*

In order to better understand the cumulative effect of known T2D-related genetic variants, genetic risk score (GRS) has been developed. For example, 22 T2D-related SNPs identified by GWAS were chosen, where 15 SNPs affect β-cell function, and 7 SNPs affect insulin response, and the number of risk alleles present for each SNP was summed as a GRS for each individual (Layton et al., 2018). The GRSs were found to be significantly related to the risk of T2D in African Americans. There were also several studies using GRS to examine the effect of SNPs on diet interactions and disease risk. In the Malmö Diet and Cancer cohort (1991–1996) in Sweden (Ericson et al., 2018), GRS and dietary risk score (DRS) were found to be associated with risk of T2D independently, and the individuals with both high GRS and DRS have the highest risk of T2D. However, no interaction was observed between GRS and dietary intakes in terms of disease risk. Likewise, the same observation was reported in the EPIC-InterAct case-cohort study restricted to Mediterranean diet (Langenberg et al., 2014). However, in United States men, a Western dietary pattern, characterized by a high intake of processed meat, red meat, refined cereals, butter, eggs and high-fat dairy products, showed a significant interaction with the GRS based on 10 T2D-associated SNPs, to increase the risk of T2D (Qi et al., 2009).

### The Effect of Dietary Intakes on Gene Expression and Epigenetic Modification

Besides interacting with genetic background, diet styles have been shown to change transcriptions related to T2D and increase the disease risk (Table 2). There are two types of transcriptome studies focusing on the gene expression change response to long-term dietary interventions or differentially expressed transcripts comparing the conditions from different habitual dietary exposures. Dietary intervention studies were usually carried out in rat or mice, and the effects on the metabolism were tested in both maternal and offspring. Low-protein diets in rat model down-regulated the expression of NR1H3 and then increased the expression of hepatic gluconeogenic genes (including G6PC and HSD11B1) and consequently resulted in glucose intolerance in adult offspring (Vo et al., 2013). Vitamin D deficiency in pregnant rat induced the down-regulation of nuclear factor κB inhibitor α (Iκbα) and resulted in insulin resistance in the offspring, which was associated with persistently increased inflammation (Zhang et al., 2014). Chromium was reported to regulate blood glucose first in 1959 (Mertz and Schwarz, 1959), a recent study using a mouse model found that chromium deficiency increased T2D susceptibility by downregulating insulin signaling genes to result in glucose intolerance (Zhang et al., 2017).

**TABLE 2:** Dietary intakes changing gene expression and epigenetics increase the risk of type 2 diabetes.

Dietary factors	Target genes	Modification	References
Low protein	NR1H3	Low expression	Vo et al., 2013
Low protein	HSD11B1, G6PC	High expression	Vo et al., 2013
Vitamin D deficiency	NFKBIA	Low expression	Zhang et al., 2014
Chromium deficiency	Insulin signaling genes	Low expression	Zhang et al., 2017
Low protein	NR1H3	Histone acetylation decrease	Vo et al., 2013
Vitamin D deficiency	NFKBIA	DNA methylation	Zhang et al., 2014
Chromium deficiency	Insulin signaling genes	DNA methylation	Zhang et al., 2017
Magnesium deficiency	HSD11B2	DNA unmethylation	Takaya et al., 2011
Calcium deficiency	HSD11B1	DNA methylation	Takaya et al., 2013

*NR1H3, nuclear receptor subfamily 1 group H member 3; HSD11B1, hydroxysteroid 11-β dehydrogenase 1; G6PC, glucose-6-phosphatase; NFKB1, nuclear factor κB subunit 1; HSD11B2, hydroxysteroid 11-β dehydrogenase 2.*

Epigenetics, including DNA methylation, histone modification, non-coding RNAs, chromatin structure, and so on, can regulate gene expression without changing the DNA coding sequence. Epigenetics are inheritable and reversible processes and involved in every aspect of life, for example, cell differentiation, embryogenesis, and development. In recent years, researches have recovered that epigenetic changes play an important role in various diseases including cancers, mental disorders, immune disease, diabetes, and cardiovascular diseases. In several population studies (Chambers et al., 2015; Dayeh et al., 2016; Wahl et al., 2017), DNA methylation markers were reported to be significantly associated with T2D incidence, and the DNA methylation risk score was able to predict the risk of T2D. The environment and lifestyle can directly interact with the genome to modify the epigenetics, and their influence can even be passed to the next generation. In the previous study, low-protein diet decreased the acetylation of histone H3 surrounding NR1H3 promoter to silence its expression and increased the risk of T2D in the offspring (Vo et al., 2013). Iκbα expression was found to be repressed potentially by Iκbα methylation when vitamin D was deficient (Zhang et al., 2014). DNA methylation profiling of the maternal liver tissue with chromium restriction diet revealed hypermethylated genes mainly involved in insulin signaling pathway; these genes were downregulated and consequently promoted T2D (Zhang et al., 2017). Likewise, magnesium and calcium deficiency increased the risk of T2D by inducing DNA methylation aberrations in genes related to glucocorticoid metabolism (Takaya et al., 2011, 2013).

### The Effect of Dietary Intakes on Gut Microbiota

Gut microbiome is related to the pathogenesis of most chronic diseases, for example, controlling body weight and regulating insulin resistance. Among the environmental factors contributing to T2D, diet plays an important role through changing the gut microbiome. With the technology advances recently, 16S rRNA gene amplicon sequencing, shotgun metagenomic, and metatranscriptomic sequencing have been well established and widely used for comprehensive mapping of gut microbes. In a recent review (Gurung et al., 2020), the authors summarized 42 observational studies about bacterial microbiome and T2D and reported that five genera (including *Bifidobacterium*, *Bacteroides*, *Faecalibacterium*, *Akkermansia*, and *Roseburia*) were negatively associated with T2D, whereas the genera of *Ruminococcus*, *Fusobacterium*, and *Blautia* were positively associated with T2D. It is known that gut microbiota influences the nutrition absorption, and correspondingly, nutrition modulates the composition of gut microbiota. Several literatures have studied how food intakes change the gut microbiome and then promote T2D. From 59 T2D patients, high-carbohydrate, high-fat, and high-protein diets were found to increase counts of *Clostridium* clusters IV and XI and decreased counts of *Bifidobacterium* species, order *Lactobacillales*, and *Clostridium* cluster IV in gut; therefore, fecal short-chain fatty acid (SCFA) production was decreased subsequently, leading to metabolic disorders, which increased the blood insulin levels and insulin resistance (Yamaguchi et al., 2016).

These scientific advances allow us to predict individual risk by taking into account the genetic, epigenetic information, and dietary habits, thus enabling personalized prevention of the disease by formulating dietary recommendations.

## Diet With Genetics, Epigenetics, and Metagenomics Involved in the Intervention of T2D

Dietary intervention is an important way to control blood glucose levels in the treatment of T2D. There have been more recognitions that nutrition adjustment for T2D, which mainly aim at adjusting the metabolic disorders (i.e., insulin resistance), has different responses, given the individuals’ genetic features. Dietary interventions can also change the expression and epigenetic feature of genes involved in the important metabolic pathway, whereas the expression profiles and epigenetic markers can be used to predict personalized response. Moreover, the gut microbiota compositions can be modulated directly by nutrition during dietary interventions.

### SNPs–Diet Interactions Showing Differential Responses to Dietary Intervention

FTO rs1558902 polymorphisms with high-fat diet were reported to improve the insulin sensitivity differently rather than low-fat diet from a randomized weight-loss dietary interventional trial (Zheng et al., 2015). Similarly, food interventions aimed at restricting caloric intake or modifying energy derived from fat, protein, or carbohydrates were screened with several SNPs, showing that different SNPs–diet interactions resulted in varied response in terms of weight loss, fasting insulin, and HOMA-IR (Grau et al., 2010; Qi et al., 2011, 2012, 2015; Xu et al., 2013; Huang T. et al., 2015). In a POUNDS LOST trial (Huang et al., 2016), a 2-year low-protein weight-loss diet for individuals with low diabetes GRS was found to significantly improve β-cell function and insulin resistance, whereas a high-protein diet might be more beneficial for patients with high GRS.

### Epigenetic Modification After Dietary Intervention

Epigenetics play an important role in the metabolic disorders contributing to T2D, whereas lifestyle interventions aiming at diet and physical activity can reversely change the epigenetics and metabolic pathways. The current nutritional recommendations for diabetes management mostly aim to achieve modest weight loss and maintenance. There are not much direct studies about dietary intervention for T2D patients; however, several weight loss programs studied the interactions between DNA methylation and diet intervention. In a trial using 27 obese women with an 8-week low-calorie diet to study the interindividual difference (Cordero et al., 2011), good responders with a successful weight loss showed lower methylation of LEP and TNF-α promoter in adipose tissue and improved the lipid profile and fat mass percentage after the dietary intervention. This observation indicates the potential to predict the efficiency of weight loss by dietary intervention using DNA methylation of LEP and TNF-α promoter. Similarly, differential methylation of five regions located in or near AQP9, DUSP22, HIPK3, TNNT1, and TNNI3 genes was discovered between high and low responders to a weight loss intervention (Moleres et al., 2013). Interestingly, a pilot study reported that DNA methylation patterns of RYR1, TUBA3C, and BDNF in peripheral blood mononuclear cell were changed after weight loss intervention, and the DNA methylation pattern in the successful weight loss maintainers for up to 3 years after intervention was similar to normal-weight individuals rather than obese participants (Huang Y.T. et al., 2015). Therefore, DNA methylation markers might be used to predict body weight maintenance after weight loss.

### Modulation of Gut Microbiota After Dietary Intervention

Unhealthy food intakes, e.g., Western diet, might change gut microbiota to increase the risk of T2D; conversely, the gut microbiome can be used as a target for the treatment of T2D. Several studies have shown that dietary intervention can modulate gut microbiota composition to treat T2D (Table 3). Deficiency in SCFA production has been associated with T2D (Puddu et al., 2014). A randomized clinical study using fecal shotgun metagenomic sequencing uncovered that high-fiber diet increased the abundance of SCFA-producing microbiota in TD2 patients to alleviate their phenotype (Zhao L. et al., 2018). Similarly, fiber-rich macrobiotic Ma-Pi 2 diet or a recommended control diet for T2D treatment (Candela et al., 2016) was found to have a positive impact on modulating gut microbe dysbiosis, especially recovering the SCFA-producing microbiome such as *Faecalibacterium*, *Roseburia*, *Lachnospira*, *Bacteroides*, and *Akkermansia*. Moreover, the Ma-Pi 2 diet showed the potential to reverse proinflammatory dysbiosis in T2D by counteracting the increase in the proinflammatory groups, such as *Collinsella* and *Streptococcus*. Low-calorie formula diet was proven to have a favorable impact on gut microbiome in a standardized three-phase weight loss program for T2D patients (Taheri et al., 2019). The result showed that all of the participants lost their weight and accompanied by a significant improvement of glucose metabolism indicated by a reduction of HbA_1__c_, fasting glucose, and insulin. Meanwhile, both the phylogenetic diversity and β diversity markedly shifted during the end of the low-calorie formula diet. Based on the epidemiological studies, increased circulating BCAAs are associated with insulin resistance and T2D. A randomized crossover trial performed on T2D patients identified that decreased intake of BCAAs was negatively relevant to postprandial insulin secretion (Karusheva et al., 2019). Meanwhile, the analysis of fecal microbiome showed enrichment in *Bacteroidetes* but decrease of *Firmicutes*.

**TABLE 3:** Dietary interventions modulate gut microbiota.

Dietary factors	Microbiota modulation	Metabolism	References
Fiber-rich diet	Higher counts of SCFA-producing gut microbiota	Improving SCFA production	Zhao L. et al., 2018
Ma-Pi 2 diet	Higher counts of SCFA-producing microbiota	Improving SCFA production	Candela et al., 2016
Low-calorie diet	Increased gut microbiota diversity, reducing levels of *Collinsella*		Taheri et al., 2019
Branched-chain amino acids reduced diet	Enrichment in *Bacteroidetes* but decrease of *Firmicutes*	Reducing meal-induced insulin secretion	Karusheva et al., 2019
Pumpkin polysaccharide	Enrichment of the butyric acid–producing gut microbiota	Improving SCFA production	Liu et al., 2018
Combination of aronia, red ginseng, shiitake mushroom, and nattokinase	Improving gut microbiome dysbiosis	Enhancing insulin secretion and reducing insulin resistance	Yang et al., 2018
Oil tea	Higher counts of Lachnospiraceae		Lin et al., 2018
Red pitaya β-cyanins	Decreasing the ratio of *Firmicutes* and *Bacteroidetes*, increasing relative abundance of *Akkermansia*	Improving insulin resistance	Song et al., 2016
*Lessonia nigrescens* ethanolic extract	Enrichment of beneficial bacteria, *Barnesiella*, decrease of *Clostridium* and *Alistipes*	Higher expression of PI3K and lower expression of JNK in liver	Zhao C. et al., 2018

*PI3K, phosphatidylinositol 3-kinase; JNK, c-Jun N-terminal kinase.*

There were also many researches using animal models to investigate dietary intervention for T2D. A report demonstrated that pumpkin polysaccharide had the ability to ease the phenotype of T2D in rat model induced by high-fat diet and streptozotocin and selectively enriched the butyric acid–producing microbiota in rat gut, which is the potential mechanism (Liu et al., 2018). In a non-obese type 2 diabetic animal model, the pancreatectomized rats were provided diets supplemented with aronia, red ginseng, shiitake mushroom, and nattokinase (AGM) (Yang et al., 2018). After 12 weeks’ feed, the experimental group showed enhanced insulin secretion and reduced insulin resistance, as well as improved the gut microbiome dysbiosis. According to traditional Chinese medicine, oil tea containing green tea and ginger has potential to treat various ailments (Lin et al., 2018). Lin et al. (2018) orally gavaged the *db/db* mice with oil tea for 8 weeks and tested FBG, oral glucose tolerance test, and lipid levels. The result showed that oil tea can effectively suppress the blood glucose elevation, and meanwhile the gut microbiota was markedly enriched with Lachnospiraceae. It was also reported that the high-fat diet–fed mice showed reduced insulin resistance after oral gavage red pitaya β-cyanins for 14 weeks (Song et al., 2016). 16S rRNA sequencing analysis found the structure of gut microbiota was modulated especially with the decreased ratio of *Firmicutes* and *Bacteroidetes* and increased relative abundance of *Akkermansia*. In another streptozotocin-induced type 2 diabetic mice model, *Lessonia nigrescens* ethanolic extract was shown to decrease FBG levels (Zhao C. et al., 2018). The gene and protein of PI3K in liver were upregulated, whereas JNK was significantly downregulated. Meanwhile, the gut microbiota was enriched with beneficial bacteria, *Barnesiella*, and had less abundances of *Clostridium* and *Alistipes*.

In summary, previous studies have shown that the specific dietary (e.g., high-fiber, low-fat, or low-calorie formula diet) can regulate insulin secretion and resistance through modulating the gut microbiota. Therefore, modification of the gut bacteria composition by dietary intervention might be a feasible method to alleviate the symptom of T2D. However, concrete conclusion remains to be obtained by future well-designed and long-term studies. Especially, more effort is expected to study the role of individual food compounds or nutrients in regulating the metabolism to prevent and treat T2D.

## Conclusion

Type 2 diabetes is a metabolic disease characterized by insulin resistance and/or abnormal insulin secretion that is caused by multiple factors including genetics, nutrition, and physical activity. Insulin signaling dysregulation in glucose metabolism is the major mechanism contributing to T2D, and the factors involved in this pathway can be targets for prevention and intervention of T2D. Growing evidence suggest the important role of nutrition in developing T2D, and the mechanisms behind are explored, respectively, in terms of five main nutrients, namely, proteins, carbohydrates, fats, vitamins, and minerals. Previous suggestions for management of T2D are usually made based on average population; however, with the advance of precision medicine, precision nutrition has attracted increasing attention in T2D. Genetic predisposition combined with diet specifically influences the risk of developing T2D for individuals, and food intakes change gene expression, epigenetic features, and gut microbiota to characterize individuals’ response to prevention and treatment by adjusting dietary patterns. However, precision nutrition is still in its infancy, and the studies performed are not comprehensive and sometimes have contradicted conclusions, possibly due to the limited sample size, varied population, and unstandardized study design. In conclusion, nutrition plays a big role in the prevention and intervention of T2D, and precision nutrition holds promise for future therapeutic strategies.

## Author Contributions

YG and QL conceived the idea and wrote the manuscript with input from ZH, DS, and QG. YG prepared the figures. QL and ZH prepared the tables. All authors edited and approved the final manuscript.

## Conflict of Interest

The authors declare that the research was conducted in the absence of any commercial or financial relationships that could be construed as a potential conflict of interest.
